# Rigorous optimisation of multilinear discriminant analysis with Tucker and PARAFAC structures

**DOI:** 10.1186/s12859-018-2188-0

**Published:** 2018-05-30

**Authors:** Laura Frølich, Tobias Søren Andersen, Morten Mørup

**Affiliations:** Department of Applied Mathematics and Computer Science, Technical University of Denmark, Building 324, Kongens Lyngby, 2800 Denmark

**Keywords:** Multilinear discriminant analysis, Electroencephalography, EEG, Tensor, Classification, Stiefel manifold

## Abstract

**Background:**

We propose rigorously optimised supervised feature extraction methods for multilinear data based on Multilinear Discriminant Analysis (MDA) and demonstrate their usage on Electroencephalography (EEG) and simulated data. While existing MDA methods use heuristic optimisation procedures based on an ambiguous Tucker structure, we propose a rigorous approach via optimisation on the cross-product of Stiefel manifolds. We also introduce MDA methods with the PARAFAC structure. We compare the proposed approaches to existing MDA methods and unsupervised multilinear decompositions.

**Results:**

We find that manifold optimisation substantially improves MDA objective functions relative to existing methods and on simulated data in general improve classification performance. However, we find similar classification performance when applied to the electroencephalography data. Furthermore, supervised approaches substantially outperform unsupervised mulitilinear methods whereas methods with the PARAFAC structure perform similarly to those with Tucker structures. Notably, despite applying the MDA procedures to raw Brain-Computer Interface data, their performances are on par with results employing ample pre-processing and they extract discriminatory patterns similar to the brain activity known to be elicited in the investigated EEG paradigms.

**Conclusion:**

The proposed usage of manifold optimisation constitutes the first rigorous and monotonous optimisation approach for MDA methods and allows for MDA with the PARAFAC structure. Our results show that MDA methods applied to raw EEG data can extract discriminatory patterns when compared to traditional unsupervised multilinear feature extraction approaches, whereas the proposed PARAFAC structured MDA models provide meaningful patterns of activity.

**Electronic supplementary material:**

The online version of this article (10.1186/s12859-018-2188-0) contains supplementary material, which is available to authorized users.

## Background

Linear Discriminant Analysis (LDA) is a widely used method for feature extraction, dimensionality reduction, and classification [1, 2]. When the number of observations is substantially larger than the number of observed variables, LDA often obtains high classification rates ([2], p. 111), especially taking its relatively simple formulation and estimation into account. However, there are cases in which each observed entity is not a vector, but rather a matrix or a higher-order array (tensor), for example EEG data [3–6]. A tensor can be seen as a generalisation of a matrix such that a first-order tensor is a vector and a second-order tensor is a matrix. The term “mode” is important when describing a tensor, and the number of modes corresponds to the order of the tensor. In a matrix, i.e. a second-order tensor, the row number increases along the first mode while the column number increases over the second mode. The simplest way to handle higher order data is to vectorise it. However, this may lead to observation vectors longer than the number of observations. In such situations, LDA runs into singularity problems. Instead, the intrinsic multilinear structure can be retained and analysed.This is the aim of Multilinear Discriminant Analysis (MDA) methods which leverage the multilinear structure in order to find discriminatory subspaces. Unfortunately, current MDA approaches [4–14] are based on heuristic optimisation approaches that do not rigorously optimise the MDA objectives according to the imposed multilinear structure. In particular, they do not maintain the desired Tucker structure and constraints on interactions between modes throughout the optimisation, but resort to alternating heuristics.

### Contributions

In this paper, we set out to investigate:

*What are the gains from optimising MDA rigorously over existing alternating heuristics?*

We investigate whether rigorous optimisation on the cross-product of Stiefel manifolds results in better solutions as quantified by the MDA objective function and classification performance than the existing heuristic optimization procedures. In particular, we consider trace-ratio optimisation of matrices and compare them to existing trace-ratio optimisation procedures that have been used for MDA. We note that other procedures for optimising the trace-ratio exist [15, 16]. However, none of these procedures incorporate the cross-product of Stiefel manifolds structure of matrices presently considered.

*Is the more flexible Tucker structure necessary in MDA or does the PARAFAC structure suffice?*

While the Tucker models are subject to rotational invariance, the PARAFAC structure is more constrained and may thereby provide unique representations, making interpretation of the PARAFAC model more meaningful. We consider MDA with the PARAFAC structure, which is not possible with the existing MDA optimisation methods. For completeness, we further consider the logistic regression framework proposed in [3], both with the originally described PARAFAC structure and with the Tucker structure.

*How do classification performances using features extracted by MDA compare to features extracted using unsupervised multilinear decompositions?*

When extracting features via supervised methods, it is only possible to use observations whose class is known. On the other hand, unsupervised feature extraction methods learn from all available data, regardless of whether observations’ classes are known. Hence, if features extracted via unsupervised methods are as informative as those extracted in a supervised manner, then the features used for classification can be learned based on all data, making them more robust. This makes it relevant to investigate whether the use of labels during feature extraction yields substantially better classification results. To investigate the utility of MDA over existing unsupervised multilinear feature extraction approaches, we compare the performance of features extracted via MDA to the classification rates obtained when features are extracted using unsupervised multilinear decomposition approaches; PARAFAC [17, 18], PARAFAC2 [19, 20], Tucker, and Tucker2 [21]. In effect, we compare to previously proposed approaches with an unsupervised step followed by a supervised step [22–30].

## Methods

### Multilinear Discriminant Analysis

For clarity of exposition, we limit our presentation to matrix observations. Let ${\bar {\mathbf {X}}}$ be the mean of all *N* observations and ${\bar {\mathbf {X}}}_{c}$ be the mean of observations from class *c*. The operator *vec*(**X**) vectorises the matrix **X** column-wise.

Similar to the objective of LDA, that is, to find projections that optimally discriminate between vector observations from different classes, the objective of Multilinear Discriminant Analysis (MDA) is to find mode-specific projections that optimally separate tensor observations from different classes. Hence, MDA aims to find projection matrices that project tensor observations  into a maximally discriminative lower dimensional representation,  with *K*_*p*_≤*J*_*p*_, *p*=1,2,…,*P*. The projection matrix for mode *p* thus has the dimensions *J*_*p*_×*K*_*p*_.

We generalise the within- and between-class scatter matrices from LDA to matrix observations, respectively:

1 $$\begin{array}{*{20}l} \mathbf{W} &= \sum_{c=1}^{C}\sum_{n \in \mathcal{C}_{c}}vec\left(\mathbf{X}_{n}-{\bar{\mathbf{X}}}_{c}\right) vec\left(\mathbf{X}_{n}-{\bar{\mathbf{X}}}_{c}\right)^{\top}  \\ \mathbf{B} &=\sum_{c=1}^{C} N_{c} vec \left({\bar{\mathbf{X}}}_{c} - {\bar{\mathbf{X}}}\right) vec\left({\bar{\mathbf{X}}}_{c} - {\bar{\mathbf{X}}}\right)^{\top}. \end{array} $$

These can be generalised to general tensors, $\mathcal {X}_{n}$, by substituting all occurrences of the matrices **X**_*n*_, ${\bar {\mathbf {X}}}_{c}$, and ${\bar {\mathbf {X}}}$ by their tensor counterparts $\mathcal {X}_{n}$, $\mathcal {\bar {X}}_{c}$, and $\mathcal {\bar {X}}$.

By substituting the projection matrix in standard LDA by the Kronecker product **U**=**U**^(2)^⊗**U**^(1)^, the objective function used in LDA becomes directly applicable to matrix observations. The Kronecker product repeats the second matrix as many times as there are elements in the first matrix, scaling each repetition by the corresponding element in the first matrix [31]. A further generalisation to observations with *P* modes is straight-forward by defining **U**=**U**^(*P*)^⊗**U**^(*P*−1)^⊗…⊗**U**^(1)^. This expression of the projection matrix **U** makes it clear that it lies on a cross-product manifold, with each mode-specific projection matrix corresponding to one of the manifold factors in the cross-product. These individual manifolds determine the constraints on each projection matrix. The Stiefel manifold contains the set of all matrices whose columns are mutually orthogonal, i.e. $\mathbf {U}^{(P)^{\top }}\mathbf {U}^{(P)}=\mathbf {I}$. Hence, orthogonality constraints are enforced on all modes by optimising over a cross-product of Stiefel manifolds. Existing MDA methods [7–13] ignore this cross-product manifold structure, and most optimise mode-specific projection matrices one at a time using alternating optimisation heuristics between the modes.

Once optimal projection matrices for each mode are found, an observation, $\mathcal {X}_{n}$, can be projected into the vector $\pmb {y}_{n} = \left (\mathbf {U}^{(P)}\otimes \mathbf {U}^{(P-1)} \otimes \ldots \otimes \mathbf {U}^{(1)}\right)^{\top } vec(\mathcal {X}_{n})$, where $\pmb {y}_{n} = vec(\mathcal {Y}_{n})$. The elements in *y*_*n*_ may be given as input to a classification algorithm, e.g. logistic regression. In the case that we focus on, where each observation is a matrix, the projection to the lower-dimensional space can be written: **Y**_*n*_=**U**^(1)⊤^×**X**_*n*_**U**^(2)^. Notice that the element in row *i* and column *j* of **Y**_*n*_ gives the strength of the interaction between factor (column) *i* from mode 1 (**U**^(1)^) and factor (column) *j* from mode 2 (**U**^(2)^). When all elements of **Y**_*n*_ are allowed to be non-zero, we refer to the MDA model as having the Tucker structure. It is natural to also consider a structure in which each factor only interacts with one factor in the other modes. This is enforced by only allowing diagonal elements of **Y**_*n*_ to be non-zero, and we refer to such MDA models as having the PARAFAC structure. In such models, the *i*^*th*^ columns of all projection matrices can be viewed as expressing how a discriminative pattern for classification is expressed in each mode. A consequence of an algebraic operation necessary for the existing heuristic optimisation methods is that the existing MDA methods implement the Tucker structure and do not allow for the PARAFAC structure.

#### Heuristic solutions to multilinear discriminant analysis

The methods Discriminant Analysis with TEnsor Representation (DATER) [8] and Constrained Multilinear Discriminant Analysis (CMDA) [11] aim to optimise the “scatter ratio” objective function [8, 11] (see Eq. 5). Another existing MDA method [13] is similar to DATER, but solves the Generalised Eigenvalue problem during optimisation instead of the standard formulation. We refer to this method as DATEReig. All three methods are based on an alternating optimisation procedure estimating each mode iteratively. When updating mode *p*, they project **W** and **B** unto all modes except mode *p*:

2 $$\begin{array}{*{20}l} \mathbf{W}_{proj}^{\tilde{p}} &= \sum_{c=1}^{C}\sum_{n \in \mathcal{C}_{c}} \left(\mathbf{X}_{n}-{\bar{\mathbf{X}}}_{c}\right)_{(p)}\mathbf{U}^{\tilde{p}\top} \mathbf{U}^{\tilde{p}} \left(\mathbf{X}_{n}-{\bar{\mathbf{X}}}_{c}\right)_{(p)}^{\top}  \\ \mathbf{B}_{proj}^{\tilde{p}} &=\sum_{c=1}^{C} N_{c} \left({\bar{\mathbf{X}}}_{c} - {\bar{\mathbf{X}}}\right)_{(p)}\mathbf{U}^{\tilde{p}\top} \mathbf{U}^{\tilde{p}} \left({\bar{\mathbf{X}}}_{c} - {\bar{\mathbf{X}}}\right)_{(p)}^{\top}, \end{array} $$

where $\mathbf {U}^{\tilde {p}} = \mathbf {U}^{(P)}\otimes \ldots \mathbf {U}^{(p+1)} \otimes \mathbf {U}^{(p-1)} \ldots \mathbf {U}^{(1)}$. Note, that **X**_(*p*)_ denotes matricisation along mode *p*.

CMDA then updates **U**^(*p*)^ by setting it equal to the first *K*_*p*_ singular vectors of $\left (\mathbf {W}_{proj}^{\tilde {p}}\right)^{-1}\mathbf {B}_{proj}^{\tilde {p}}$ which was proven in [11] to result in an asymptotically bounded sequence of objective function values of the scatter-ratio objective function. Since a matrix defined by singular vectors is orthonormal, CMDA in effect uses the orthonormality constraint. DATER instead uses the first *K*_*p*_ generalised eigenvectors of the Generalised Eigenvalue Problem: $\mathbf {B}_{proj}^{\tilde {p}}\mathbf {U}^{(p)} = \mathbf {W}_{proj}^{\tilde {p}}\mathbf {U}^{(p)}\Lambda _{k}$, which leads to $\mathbf {W}_{proj}^{\tilde {p}}$-orthogonality ($\mathbf {U}^{(p)^{\top }}\mathbf {W}_{proj}^{\tilde {p}}\mathbf {U}^{(p)}=\mathbf {\Lambda }$, where **Λ** is a diagonal matrix [32]). Since the matrix $\mathbf {W}_{proj}^{\tilde {p}}$ is different for each mode, this means that the projection matrices for the different modes are constrained differently by DATER. DATEReig instead solves the Standard Eigenvalue Problem, defined as: $\left (\mathbf {W}_{proj}^{\tilde {p}}\right)^{-1}\mathbf {B}_{proj}^{\tilde {p}}\mathbf {U}^{(p)} = \mathbf {D}\mathbf {U}^{(p)}$, where **D** is a diagonal matrix. Hence DATEReig is also subject to orthonormal constraints on the projection matrices. The algorithm Higher Order Discriminant Analysis (HODA) [33] also iterates over modes in a similar fashion. HODA was seen to not be competitive on simulated data, and was not included in the comparisons on EEG data. Finally, the method Direct General Tensor Discriminant Analysis (DGTDA) [11] optimises the difference between the scatter matrices. It does this by iterating over each mode once, independently for each mode without projection, setting *ζ* equal to the largest singular value of (**W**_(*p*)_)^−1^**B**_(*p*)_ when solving for mode *p*. The projection matrix for mode *p* is then set equal to the first *K*_*p*_ singular vectors of **B**_(*p*)_−*ζ***W**_(*p*)_.

Rather than optimising a measure of class-separability, it may be advantageous to optimise classification performance directly. Bilinear Discriminant Component Analysis (BDCA) implements this idea through logistic regression with a PARAFAC structure [3, 34]. The log-likelihood for BDCA is:

3 $$ \begin{aligned} {}\sum_{n=1}^{N}y_{n}&(w_{0}+\psi_{PARAFAC}(\mathbf{X}_{n})) \\ &- \log(1+\exp(w_{0}+\psi_{PARAFAC}(\mathbf{X}_{n})), \end{aligned}  $$

such that the probability that observation **X**_*n*_ belongs to class one is $ \frac {1}{1+\exp \left (-\left (w_{0}+\psi _{PARAFAC}\left (\mathbf {X}_{n}\right) \right)\right)}$, where

$$\begin{aligned} {}\psi_{PARAFAC}(\mathbf{X}_{n})&=Tr\left(\mathbf{U}^{(1)^{\top}} \mathbf{X}_{n}\mathbf{U}^{(2)}\right)\\ &= \sum_{k=1}^{K_{1}}\left[\left(\mathbf{U}^{(1)}\odot \mathbf{U}^{(2)}\right)^{\top} vec(\mathbf{X}_{n})\right]_{k}. \end{aligned} $$

Thus, the number of components is the same in both modes (*K*_1_=*K*_2_) and there are no constraints on the projection matrices. Despite the PARAFAC type of structure, the model is not unique. For two square matrices satisfying $\phantom {\dot {i}\!}\mathbf {Q}^{(2)}\mathbf {Q}^{(1)^{\top }}=\mathbf {I}$, we have:

$$\begin{aligned} {}&Tr\left(\left(\mathbf{U}^{(1)}\mathbf{Q}^{(1)}\right)^{\top} \mathbf{X}_{n}\left(\mathbf{U}^{(2)}\mathbf{Q}^{(2)}\right)\right)\\& =Tr\left(\mathbf{Q}^{(2)}\mathbf{Q}^{(1)^{\top}}\mathbf{U}^{(1)^{\top}} \mathbf{X}_{n}\mathbf{U}^{(2)}\right) = Tr\left(\mathbf{U}^{(1)^{\top}} \mathbf{X}_{n}\mathbf{U}^{(2)}\right), \end{aligned} $$

hampering model interpretation unless additional constraints are imposed.

For comparison, we introduce a Tucker-structure version of the above logistic regression model, resulting in the following log-likelihood:

$$\begin{array}{*{20}l} {}\sum_{n=1}^{N}y_{n}\!\left(w_{0}+\psi_{Tucker}(\mathbf{X}_{n})\right)  \,-\, \log(1\,+\,\exp\!\left(w_{0}\,+\,\psi_{Tucker}\left(\mathbf{X}_{n}\right)\right), \end{array} $$

where

$$\begin{array}{*{20}l} \psi_{Tucker}(\mathbf{X}_{n}) = \sum_{k_{1}=1}^{K_{1}}\sum_{k_{2}=1}^{K_{2}} \left[\mathbf{U}^{(1)\top} \mathbf{X}_{n} \mathbf{U}^{(2)}\right]_{{k_{1}},{k_{2}}} \mathbf{V}_{k_{1},k_{2}}, \end{array} $$

with $\mathbf {V}_{{k_{1}},{k_{2}}}=1$ for *k*_1_=*k*_2_ to remove scaling ambiguities between the projection matrices and the matrix of interaction coefficients, **V**. As for BDCA, there are no constraints on **U**^(1)^ and **U**^(2)^.

### MDA based on manifold optimisation with PARAFAC and Tucker structures

The existing MDA approaches rely on heuristic optimisation procedures based on either eigenvalue or singular value decompositions. Instead, we propose to exploit the manifold optimisation in the recently released *ManOpt* toolbox [35]. This toolbox implements rigorous optimisation of arbitrary objective functions on a variety of manifolds, as long as their gradients are known. Amongst others, the toolbox has implementations of optimisation over the Stiefel manifold, which consists of orthonormal matrices [36]. By optimising over a cross-product of Stiefel manifolds, one for each mode, all projection matrices are optimised simultaneously under orthonormality constraints. Notably, other constraints can be enforced on some or all modes by changing the manifolds in the cross-product manifold.

We propose four new MDA methods by optimising the scatter ratio objective [8, 11] and three new MDA objective functions rigorously. We impose orthonormality constraints through optimisation on a cross-product of Stiefel manifolds and optimise the model parameters using the conjugate gradient method. The three new objective functions are a PARAFAC version of the scatter ratio objective and a PARAFAC and Tucker version of the trace-ratio objective [1].

The orthonormal projection matrices with the Tucker and PARAFAC structures are defined through the Kronecker and Khatri-Rao products, respectively:

4 $$\begin{array}{*{20}l} \mathbf{U}_{Tucker} &= \mathbf{U}^{(P)}\otimes \mathbf{U}^{(P-1)}\ldots \mathbf{U}^{(1)} \\ \mathbf{U}_{PARAFAC} &= \mathbf{U}^{(P)}\odot \mathbf{U}^{(P-1)}\ldots \mathbf{U}^{(1)}. \end{array} $$

The Khatri-Rao product is the column-wise Kronecker product [31]. The objective functions and the names we refer to the methods by are:

Manifold Tucker/PARAFAC Discriminant Analysis with the scatter ratio objective (ManTDA_sr/ ManPDA_sr):

5 $$\begin{array}{*{20}l} \frac{Tr\left(\mathbf{U}_{s}^{\top} \mathbf{B} \mathbf{U}_{s}\right)}{Tr\left(\mathbf{U}_{s}^{\top} \mathbf{W} \mathbf{U}_{s}\right)}. \end{array} $$

Manifold Tucker/PARAFAC Discriminant Analysis with the trace of matrix ratio objective (ManTDA/ManPDA):

6 $$\begin{array}{*{20}l} Tr\left(\left(\mathbf{U}_{s}^{\top}\mathbf{W}\mathbf{U}_{s}\right)^{-1} \mathbf{U}_{s}^{\top} \mathbf{B} \mathbf{U}_{s}\right), \end{array} $$

where the structure variable *s* is either *Tucker* or *PARAFAC*. Another proposed objective function uses determinants [13, 37]. The solution to this objective has the same stationary points as (6) (see Additional file 1: Appendix A).

While the scatter ratio objective (5) maximises the ratio of energy in between-class observations relative to within-class observations, the trace of matrix ratio objective (6) maximises the ratio of the volume spanned by between-class observations to the volume spanned by within-class observations.

### Logistic regression for classification

For all methods, we use logistic regression for classification. For the MDA methods, discriminative projections are first found, and then used to project observations onto low-dimensional spaces, and the scalar values in these representations (matrices) are used for classification. For BDCA and BDCATucker, the logistic regression classification step is an integral part of the method. For the unsupervised methods (PARAFAC, PARAFAC2, Tucker, and Tucker2), data is decomposed and the estimated factors for the trial mode for each observation are used as features for logistic regression. While logistic regression is perhaps the most simple classifier, we use it to compare the degree of linear separability of classes obtained using each of the methods.

### Uniqueness of MDA

MDA based on the Tucker structure is not unique when considering the objective functions given above. In fact, the projection matrix for each mode can separately be multiplied by any orthonormal matrix ***R*** without changing the value of the objective function, as shown in the (Additional file 2: Appendix B).

For the PARAFAC version of MDA (for *P*=2) we can consider alternative representations of **U**=**U**^(2)^⊙**U**^(1)^ by multiplying two orthonormal matrices **R**^(1)^ and **R**^(2)^ to form $\tilde {\mathbf {U}}=\left (\mathbf {U}^{(2)} \mathbf {R}^{(2)}\right)\odot \left (\mathbf {U}^{(1)}\mathbf {R}^{(1)}\right)$. Exploiting the property [38]:

$$\begin{array}{*{20}l} {}\left(\mathbf{U}^{(2)} \mathbf{R}^{(2)}\right)\odot \left(\mathbf{U}^{(1)}\mathbf{R}^{(1)}\right)=\left(\mathbf{U}^{(2)} \otimes \mathbf{U}^{(1)}\right)\left(\mathbf{R}^{(2)}\odot \mathbf{R}^{(1)}\right), \end{array} $$

we obtain for the term used separately in the numerator and denominator of the scatter ratio objective function (5):

$$\begin{array}{*{20}l} \tilde{\mathbf{U}} \tilde{\mathbf{U}}^{\top}&= \left(\mathbf{U}^{(2)} \otimes \mathbf{U}^{(1)}\right)\left(\mathbf{R}^{(2)}\odot \mathbf{R}^{(1)}\right)\\ &\quad\,\,\left(\mathbf{R}^{(2)}\odot \mathbf{R}^{(1)}\right)^{\top}\left(\mathbf{U}^{(2)} \otimes \mathbf{U}^{(1)}\right)^{\top}, \end{array} $$

and for the trace of matrix ratio objective (6):

7 $$ {\selectfont{\begin{aligned} {}Tr\left(\left(\tilde{\mathbf{U}}^{\top}\mathbf{W}\tilde{\mathbf{U}}\right)^{-1} \tilde{\mathbf{U}}^{\top} \mathbf{B} \tilde{\mathbf{U}}\right) \!=& Tr\left(\left(\left(\mathbf{R}^{(2)}\!\odot\! \mathbf{R}^{(1)}\right)^{\top}\!\left(\mathbf{U}^{(2)} \!\otimes\! \mathbf{U}^{(1)}\right)^{\top}\!\mathbf{W}\right.\right.\\ &\left.\left(\mathbf{U}^{(2)} \otimes \mathbf{U}^{(1)}\right)\left(\mathbf{R}^{(2)}\odot \mathbf{R}^{(1)}\right)\right)^{-1} \\ & \left(\mathbf{R}^{(2)}\odot \mathbf{R}^{(1)}\right)^{\top}\left(\mathbf{U}^{(2)} \otimes \mathbf{U}^{(1)}\right)^{\top}\mathbf{B}\\ & \left.\left(\mathbf{U}^{(2)} \otimes \mathbf{U}^{(1)}\right)\left(\mathbf{R}^{(2)}\odot \mathbf{R}^{(1)}\right)\right). \end{aligned}}}  $$

Due to the Khatri-Rao product structure it is no longer given that the above objective functions for $\tilde {\mathbf {U}}$ can be reduced to the objective functions based on **U** except for the trivial situation in which **R**^(2)^ and **R**^(1)^ are identical permutation matrices. We empirically tested the objective functions where $\mathbf {R}^{(2)}=\mathbf {R}^{(1)}, \mathbf {R}^{(2)}\,=\,\mathbf {R}^{(1)^{\top }}\phantom {\dot {i}\!}$, and $\phantom {\dot {i}\!}\mathbf {R}^{(2)}\neq \mathbf {R}^{(1)^{\top }}$ and found that the random orthonormal matrices we generated indeed did not provide equivalent objective function values. Note that the case **R**^(2)^=**R**^(1)^ would result in the same log-likelihood for *BDCA*.

## Data

In data with a temporal and a spatial mode, such as EEG data, the PARAFAC structure assumes that each spatial pattern has one associated prototypical time series, and vice versa. On the other hand, the Tucker structure allows for each spatial pattern to be active according to any of the temporal patterns, and vice versa. Depending on the phenomenon under investigation and previous knowledge, one of these assumptions on interactions between spatial and temporal patterns is likely to be more probable than the other. Hence we expect tensor models to represent probable hypotheses of EEG data generation, and compared the methods on simulated data and on two EEG data sets.

### Simulated data

We simulated one core with the Tucker structure for each of two classes. We then added noise to these cores when generating each observation. This was done by adding noise to the cores to simulate noisy realisations of the underlying cores, drawn from i.i.d. normal distributions. We then multiplied the noisy cores by simulated components to get observations in the observation space, for which we simulated observations with the dimensionionality of 10 rows and 80 columns. Finally, a non-discriminative core the same size as the discriminative core was simulated for each observation. These non-discriminative cores were multiplied by non-discriminative components, and added as structured noise consituting non-discriminative signal components shared across the two classes. The code we used for simulation is available at https://github.com/laurafroelich/tensor_classification/tree/master/code/simulation.

### Stekelenburg & Vroomen data

This data set consists of data from Experiment 2 in a set of three experiments performed and described by Stekelenburg and Vroomen [39] containing data from 16 subjects. For our analyses, we used control trials (gray box shown on computer, no sound) and non-verbal auditory trials (clapping (103-107 ms) and tapping of spoon on cup (292-305 ms), gray box on screen). Trials containing values exceeding 150 *μ*V or lower than -150 *μ*V 200 ms prior to or 800 ms after stimulus onset were removed. The baseline of trials, defined as the mean of the 200 ms before stimulus onset, were subtracted. Trials were defined as lasting from stimulus onset until 500 ms after stimulus onset. These data were recorded at 512 Hz. We balanced the trials so that there were equally many from each class (2604 trials in total over all subjects and both classes). To make leave-one-subject-out cross-validation possible, we used 50 electrodes common to all subjects.

### BCI competition data

This is Data Set II [40] from BCI competition III [41]^1^ from a P300 speller paradigm. These data were recorded from two subjects at 240 Hz from 64 electrodes and band-pass filtered during recording between 0.1-60 Hz. We extracted trials from stimulus onset until 667 ms after stimulus onset. For each subject, a training data set containing single-trial labels was available. The test data consisted of EEG recordings and the true spelled letters, but not single-trial labels.

These two data sets represent different challenges. While there are many trials in the BCI data set (15,300 per subject for training), this data set is unbalanced, with one target trial for every five non-target trials. On the other hand, we balanced the Stekelenburg&Vroomen data set but have far fewer trials for this data set.

Since compression of the temporal mode extract the temporal signature relevant to classification, we avoid pre-processing steps such as down-sampling, band-pass filtering, and spectral decomposition.

## Empirical analyses

We compared the classification performance of logistic regression using features extracted by four existing supervised tensor methods (DATER [8], DATEReig [13], CMDA [11], and DGTDA [11]) and the proposed manifold MDA approaces (ManTDA_sr, ManPDA_sr, ManTDA, and ManPDA). Standard Linear Discriminant Analysis [1] and HODA [33] were also included in the simulation study. We used logistic regression to compare the performance of features extracted using these supervised methods to features extracted by the unsupervised methods Tucker, Tucker2 [21], PARAFAC [17, 18], and PARAFAC2 [19, 20]). For comparison, we further included BDCA [3] as well as our extension of BDCA to the Tucker representation (BDCA_Tucker), both of which combine feature extraction and logistic regression in one step.

### Classification

All classifications were performed within the logistic regression framework and the Area Under the Receiver Operating Curve (AUC) ([2], Section 9.2) was used to quantify the classification performances when single-trial labels were available. To calculate the AUC, the probabilities predicted by the logistic regression models were compared to the true single-trial labels. For the BCI data, the final classification performance was evaluated as the proportion of letters spelled correctly, as in the original competition.

**Simulated data** We simulated data with three levels of signal and three components in each of two modes. The tensor decomposition methods (both the supervised and unsupervised methods) were estimated using three components.

**Stekelenburg&Vroomen data** For the Stekelenburg&Vroomen data, we used leave-one-subject-out cross-validation (CV) to estimate the between-subject performances of the models. Each subject was left out in turn to serve as test data for model evaluation, and the models were trained on the remaining 15 subjects. To see how well each model fits the training data, we inspected classification performances when the models classified trials from the 15 CV folds that they were trained on.

**BCI data** For each of the two subjects from the BCI data, we performed 5-fold CV using the training data containing single-trial labels. Each of the following steps were performed for each subject. We inspected the models’ performance both on training data (classifying trials form the four CV folds used for training) and on validation data (classifying the trials from the CV fold left out during training). We used the CV performance to choose the number of components for each model. Each model was then trained on the entire training data set using this number of components. The resulting model was applied to the test data for which single-trial labels were not available. In a final step, these single-trial classifications were used to predict the letters spelled, and these were compared to the correct letters. Hence, our results on the letter classification task are comparable to those from the competition since we did not use the test data to choose or train models, which was also the procedure in the competition.

### Number of components

The supervised tensor classification methods find projection matrices that compress multilinear observations into lower-dimensional representations. With *K* components in each mode, the size of the lower-dimensional space becomes *K*×*K* for matrix observations $\left (\mathbf {U}^{(1)^{\top }}\mathbf {X}_{n}\mathbf {U}^{(2)}\right)$, as for our data sets. Hence, each observation leads to *K*^2^ features in the lower-dimensional discriminative space. We investigated performances for one, three, and five components for the Tucker-structure projection methods (Tucker2, CMDA, DATER, DATEReig, DGTDA, ManTDA, ManTDA_sr, and BDCA_Tucker). For the PARAFAC variants of the projection methods, only the diagonal elements are used, i.e. diag $\left (\mathbf {U}^{(1)^{\top }}\mathbf {X}_{n}\mathbf {U}^{(2)}\right)$. Hence, to get the same number of features as input to logistic regression for all methods, we also included 9 and 25 components for the PARAFAC-structure methods. Likewise, the methods PARAFAC, PARAFAC2, and Tucker only yield one feature for each mode-3 component. Hence, we also estimated these models with 9 and 25 components. Note that a Tucker structured model with a core of size *K* in all *p* modes could equivalently be written as a PARAFAC structured model of rank *K*^*p*^. However, a PARAFAC model with rank *K*^*p*^ cannot be guaranteed to have an equivalent Tucker structure representation with core of size *K*^*p*^. By including the higher number of components for PARAFAC structure models, we quantify the effect of allowing the model to be at least as flexible as the Tucker representation also passing the same number of features to the classifier.

### Model implementations

We used the *nway* [42] toolbox to estimate the PARAFAC, PARAFAC2, Tucker, and Tucker2 models. These models were initialised with the best of 10 short runs, which were themselves initialised with random matrices. The BDCA methods were initialised with random normal values. The components for the trial mode (i.e., mode 3) were constrained to be orthogonal for PARAFAC and PARAFAC2. For Tucker and Tucker2, all projection matrices were constrained to be orthogonal. Due to the rotational ambiguity between the core and the projection matrices in the Tucker, the Tucker model’s fit is not impacted by these constraints. In principle, constraints are not necessary on the PARAFAC model. However, in practice, degeneracy can be an issue, which constraints preempt. Since we do not believe orthogonality constraints imposed on the spatial mode (scalp maps) or temporal mode are plausible, we chose to constrain the trial mode to be orthogonal.

The existing MDA methods (DATER, DATEReig, CMDA, HODA, and DGTDA) were optimised by Matlab code that we wrote based on the pseudo-code in the papers describing these methods [8, 11, 13, 33]. CMDA, DATER, HODA, and DATEReig were initialised with random orthogonal matrices while DGTDA does not need initialisation.

To avoid the log-likelihood from overflowing in the first iteration for the BDCA methods, the standard deviation of the initial random values for the Stekelenburg&Vroomen data was set to 0.01 while a lower value, 10^−5^, was necessary to avoid overflow for the BCI data.

Our proposed MDA methods were optimised using the *ManOpt* [35] toolbox for Matlab. The models were initialised both with random orthonormal matrices and with projection matrices obtained from short runs of CMDA. Results from the two initialisation methods were similar, so we only show the results from random initialisation.

It was originally recommended to use the Damped Newton procedure in the *immoptibox* [43] to optimise the BDCA log-likelihood objective [3]. We optimised BDCA using both the suggested Damped Newton method and the Broyden-Fletcher-Goldfarb-Shanno (BFGS) optimisation, also available in the *immoptibox*. These two optimisation methods achieved very similar classification rates. The BFGS method was slightly faster despite it only requiring gradients. We therefore used BFGS optimisation to optimise the BDCA methods.

All iterative methods were started three times and run for up to 5000 iterations or until convergence for the Stekelenburg&Vroomen data and for 1000 iterations for the BCI data. The best of the three solutions was chosen for further analysis to minimise the risk of analysing solutions from local minima. The convergence criteria used for CMDA, DATER and DATEReig were those originally proposed for CMDA and DATER [8, 11].

### Visualisation

The projection matrices found by the supervised methods act as dimension-reducing filters that maximise the class-discriminative information in the filtered data. However, such filters are not suited for visualisation for model interpretation purposes [44]. Instead, the interesting spatial properties of the estimated sources consist of how their activity is expressed on the scalp. This can be derived from the filters by pre-multiplying the data covariance matrix of electrodes onto the filter (projection) matrix if sources are assumed uncorrelated. We extrapolated this visualisation approach established for the spatial domain to the temporal domain by pre-multiplying the data covariance of temporal samples onto the temporal filter matrices to visualise the time courses of the sources. Since the MDA models with Tucker structure and BDCA are rotationally invariant, they do not have straight-forward interpretations, except in the one-component case.

On the other hand, each column in a projection matrix can only interact with one column from projection matrices for the other modes when using the PARAFAC structure. Also, we empirically observed that the PARAFAC formulations of MDA objectives were not invariant to rotations via random orthogonal matrices, making their interpretation more intuitive. For these reasons, we limit visualisations to one-component Tucker models and PARAFAC-structure MDA models.

## Results

### Classification performance on simulated data

**Fig. 1:**
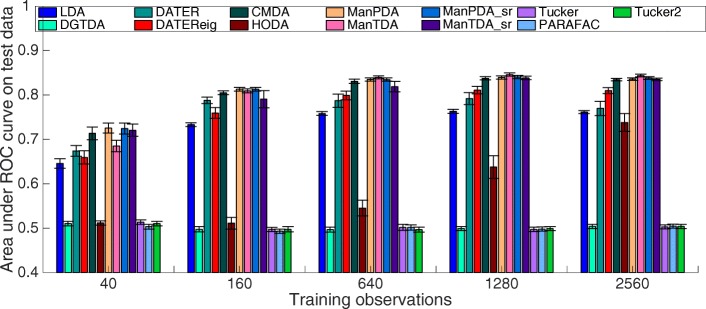
Performance on simulated data. Classification performance obtained through the tensor decomposition methods on simulated data with the medium level of simulated signal as a function of the number of training observations. Vertical lines denote plus/minus the standard deviation of the mean of 25 simulations

Figure 1 shows the classification performances of the tensor decomposition methods on simulated data with the medium level of signal strength that we simulated. The figure shows the mean AUC plus/minus the standard deviation of the mean across 25 simulations. As on the EEG data, we observe low performances from the unsupervised methods. While standard LDA outperforms the unsupervised tensor methods, the supervised tensor decomposition methods (except HODA and DGTDA) obtain higher AUCs than LDA. As expected, all methods improve with more training observations. Both DATER and DATEReig outperform LDA. CMDA is comparable in performance to the manifold methods for most numbers of training observations, but there seems to be a trend that the manifold method ManTDA is better able to leverage the addition of more training observations for large numbers of training observations. Additional plots are given in Additional file 3: Appendix C, for each of three noise levels.

### Objective function values on EEG data

**Fig. 2:**
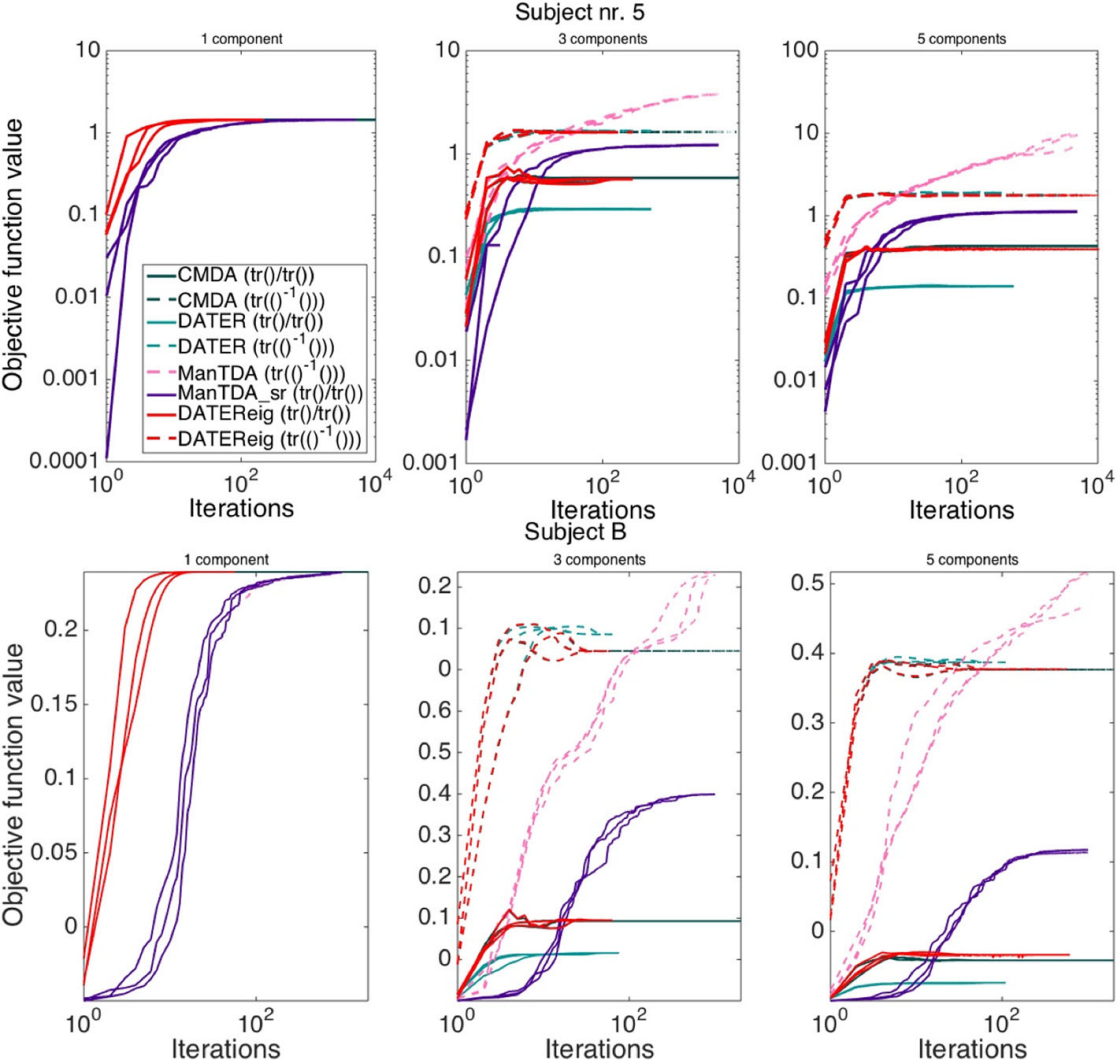
Objective function values. Objective function values for one, three, and five components. Scatter ratio objective function (5) values are shown as full lines while the matrix ratio objective (6) is shown as dashed lines for three random initialisations. *Top*: Stekelenburg&Vroomen data for the CV fold with subject 5 left out. *Bottom*: subject B from the BCI data. Note the log scale of the y-axis in the upper row and the linear scale in the bottom row

Figure 2 shows the objective function values obtained by CMDA, DATER, DATEReig, and our proposed manifold optimisation of the scatter-ratio objective with Tucker structure, the objective function these four methods aim to optimise. The values obtained for the scatter-ratio objective are shown as full lines. Objective function values for the trace of matrix ratio objective are also shown since the heuristic methods, during optimisation, use this objective as an approximation to the scatter-ratio objective. CMDA, DATEReig, and the manifold methods share the same constraints on the projection matrices and are hence directly comparable. Each iteration for DATER, DATEReig, and CMDA corresponds to an update of the projection matrix for one of the modes. Each iteration for the manifold optimisation corresponds to one update in all modes since all modes are optimised simultaneously in this approach.

The top of Fig. 2 shows a randomly chosen case of the optimisation for the CV fold with subject 5 left out in the Stekelenburg&Vroomen data. All optimisation runs were very similar to the example shown here. The bottom part of the figure shows the optimisation for CV fold number 1 for subject B. This is similar to the other CV folds, including those for subject A.

One observation from this figure is that the convergence of CMDA and DATEReig is not monotone, increasing rapidly to begin with, followed by a decline before stabilising. The alternation between optimising the two modes is seen as a sawtooth pattern of objective function values rising and falling between iterations in the initial part of the optimisation. Although more difficult to see, DATER also exhibits these characteristics. This shows that CMDA, DATEReig, and DATER do not optimise the scatter-ratio objective consistently.

Secondly, we observe that the manifold methods obtain the highest values. That is, the dashed line for ManTDA dominates the other dashed lines, while the full line for ManTDA_sr dominates the other full lines, from a certain number of iterations and onwards. DATEReig and CMDA reach the same value of the matrix ratio objective, with DATER also reaching a similar value. Since the matrix ratio is a simple, but inexact, approximation to the scatter ratio objective, it is reassuring that the iterative methods reach similar values for the inexact problem. However, their differences on the exact scatter ratio objective reveal that the inexact approximation combined with iterating over modes to optimise does not suffice to obtain the best solution to the exact problem.

### Cross-validated classification performance on EEG data

**Fig. 3:**
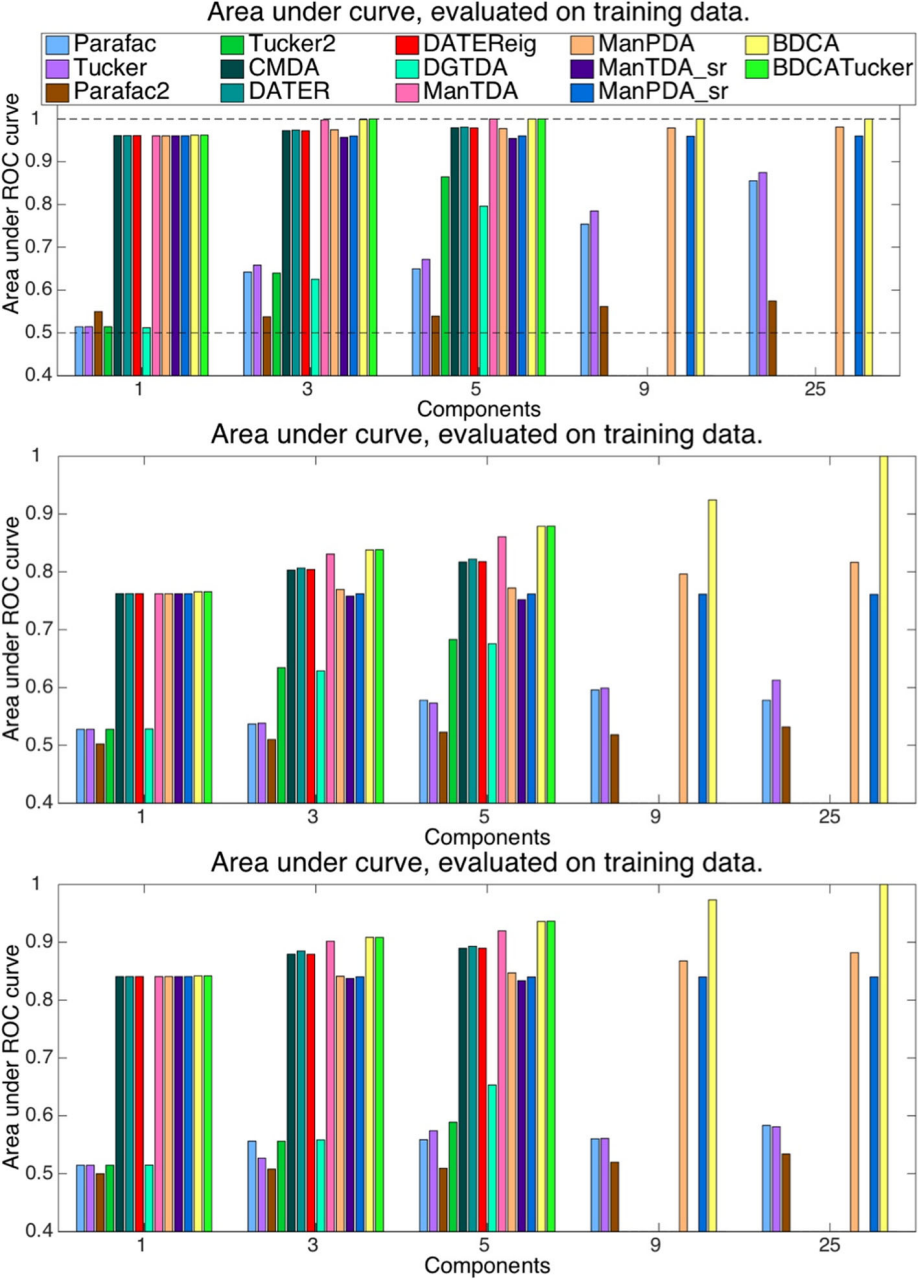
Performances on training data. Testing on training data (data from each CV fold that was also used to train on). *Top*: Stekelenburg&Vroomen data. *Middle*: BCI data, subjet A. *Bottom*: BCI data, subjet B. The methods are grouped by type such that first four methods plotted are the unsupervised decomposition methods, followed by the four heuristic supervised decomposition methods. The next four methods are the supervised manifold methods, which are followed by the two methods performing decomposition and classification in one step. Finally, the six methods that produce fewer features for classification are plotted again with 9 and 25 components

Figure 3 shows the AUC when evaluating on training data for Stekelenburg&Vroomen data (top) and for the two BCI subjects (A in the middle and B at the bottom). When evaluating on training data, all methods improved with more components, as expected.

On the Stekelenburg&Vroomen training data, ManTDA, BDCA and BDCA_Tucker outperform the other methods, even obtaining perfect classification performances (AUC value of one) whereas the other MDA methods, except DGTDA, are very close to these best performances. The PARAFAC-structure and Tucker-structure formulations of the objective functions have very similar performances but the PARAFAC-structure versions of MDA do not improve to perfection, as BDCA does for the largest component numbers. The performances are nearly identical, and low, for the unsupervised PARAFAC and Tucker models, even when allowed a large number of components. The Tucker2 method, which projects each trial into a lower dimensional space analogously to the MDA methods, performs substantially better than the other unsupervised methods, even outperforming DGTDA.

On the BCI training data, the two BDCA methods also outperform ManTDA. Here, the performance of BDCA is substantially higher than all other methods. With 25 components, BDCA again obtains AUC values of one, for both BCI subjects. On the BCI data, we observe some performance differences between ManPDA and ManTDA, with ManTDA performing best. For subject A, Tucker2 again outperforms DGTDA while it is on the same (low) level as PARAFAC and Tucker for subject B.

**Fig. 4:**
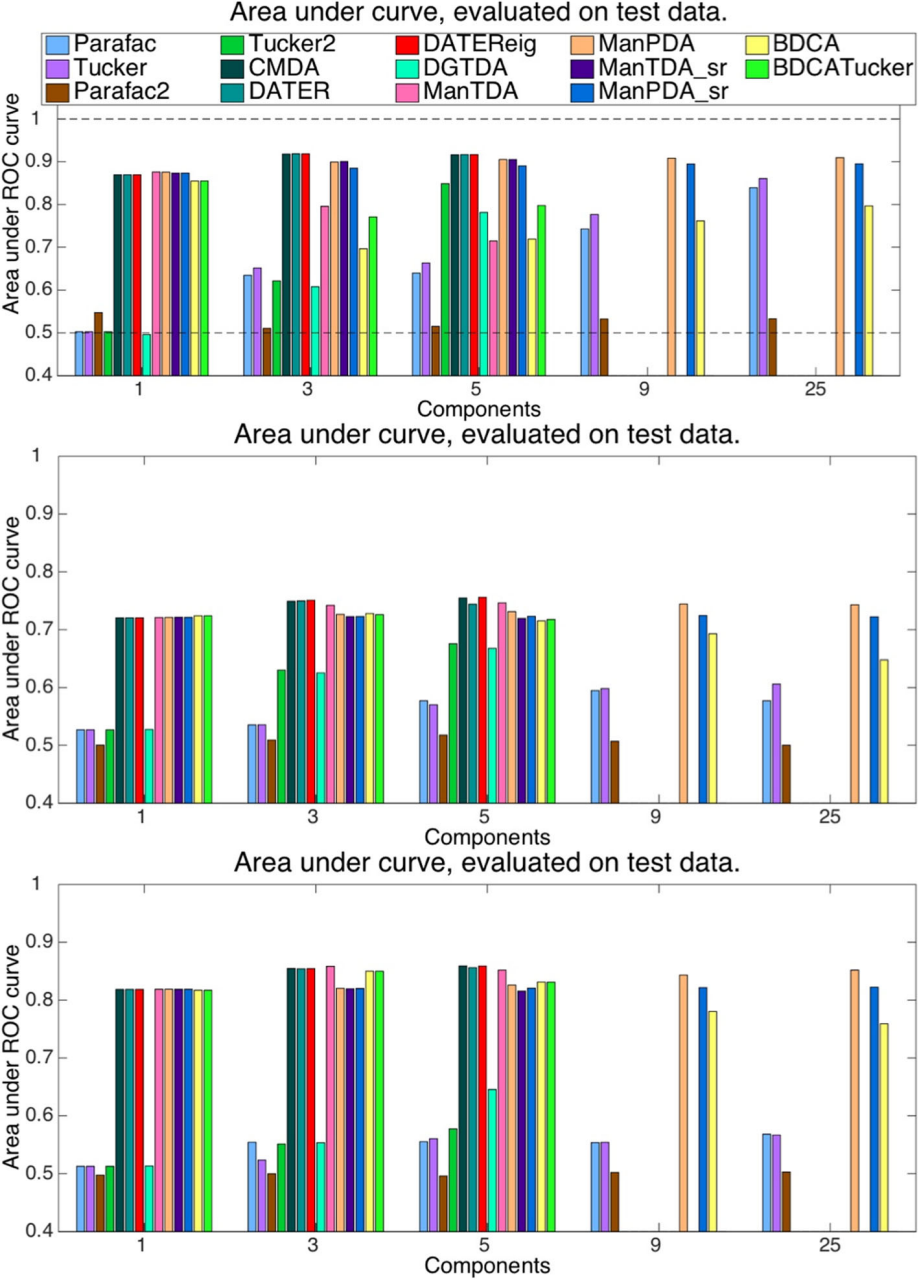
Performances on test data. Testing on validation data (data left out from each CV fold). *Top*: Stekelenburg&Vroomen data. *Middle*: BCI data, subjet A. *Bottom*: BCI data, subjet B

Figure 4 shows the classification performances obtained when evaluating on test data. Again, the results from the Stekelenburg&Vroomen data are shown in the top of the figure, with BCI subjects A and B in the middle and bottom, respectively.

When evaluating on Stekelenburg&Vroomen test data, ManTDA and the BDCA methods perform worse than the other supervised methods, especially for high component numbers. With five components, they and DGTDA are even outperformed by Tucker2. The other MDA methods still obtain the highest performances, with Tucker, PARAFAC, and PARAFAC2 only obtaining low AUCs until 25 components. At this point, Tucker and PARAFAC approach the MDA performances.

On the BCI data, ManTDA and the BDCA methods perform at the same level as the MDA methods while the unsupervised feature extraction methods do not reach this level, with any component number. With four and five components (also with three for subject A), DGTDA is somewhat better than the unsupervised methods without coming close to the other supervised methods. While the performances of CMDA, DATER, and ManTDA are slightly better, all the MDA methods perform similarly.

### BCI data letter classification performance

Table 1 shows average classification rates of letters across the two subjects in the BCI data. The first column gives the classification rates when each row/column was flashed 15 times to spell a character. The second column shows the results for five flashes. The average classification rates obtained by the five teams with highest performances in the competition are also shown, reproduced from the competition website^2^. DATEReig obtains the best performance, closely followed by CMDA, DATER and ManTDA, with only small differences between the PARAFAC and Tucker structures of the MDA methods.

### Model interpretation

We now show the temporal and spatial patterns of several of the fitted models. The components were derived and arranged in no particular order. Since the performances of the unsupervised methods are very low, we focus on visualising the supervised methods.

**Fig. 5:**
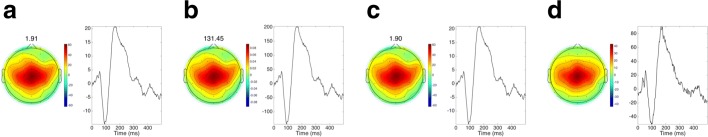
Interpretation of components from the Stekelenburg&Vroomen experiment. Spatial and temporal patterns corresponding to the extracted spatial and temporal filters found from the training data without subject 5 in the Stekelenburg&Vroomen data by the following (from top to bottom) one component models: CMDA, DATER, ManTDA, BDCA. Logistic regression coefficients are shown above the spatial patterns. **a** CMDA **b** DATER **c** ManTDA **d** BDCA

Figure 5 shows the scalp maps and corresponding temporal signatures extracted by one-component models of the Stekelenburg&Vroomen data. With only one component, the PARAFAC and Tucker versions of each objective function are identical, making BDCA and BDCA_Tucker equivalent. Also, the trace of matrix ratio is the same as the scatter ratio in this case, making all the methods optimised on manifolds equivalent. We included one-component models from each of the equivalent models in Fig. 5. Except for different scaling in DATER, the components fitted by CMDA, DATER, and ManTDA are identical. This is reflected in the nearly identical logistic regression coefficients (shown above the spatial patterns) found for CMDA and ManTDA. The magnitude of the temporal pattern found by DATER is lower than that in CMDA and ManTDA, which is accounted for by the higher logistic regression coefficient. Since the BDCA model uses the projection into a lower dimensional space directly in the logistic regression model, no coefficient is displayed for this model. Although the patterns found by BDCA are not identical to those found by the other methods, they are very similar. The temporal pattern of the component is very similar to the difference wave found by Stekelenburg and Vroomen between the two conditions [39]. The centrally located scalp map is also in good accordance with their analysis of the central Cz electrode [39]. The logistic regression model was trained to predict probabilities for the auditory class. All shown components are well in line with this training since the positive logistic regression coefficients means that centrally located scalp activity with temporal activity like the difference wave in [39] indicates that a trial is from the auditory class.

**Fig. 6:**
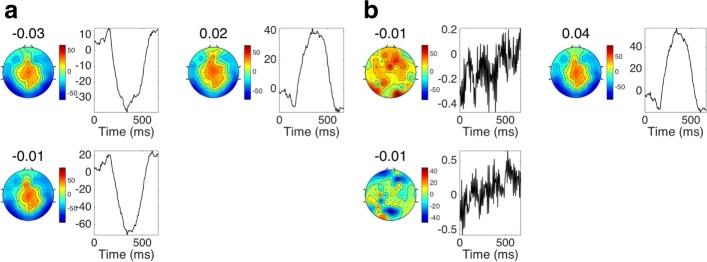
Interpretation of components from the BCI data. Spatial and temporal patterns corresponding to the extracted filters from the PARAFAC models ManPDA and ManPDA_sr, trained on four of five CV folds from subject A’s BCI data. Fitted logistic regression coefficients are shown above the spatial patterns. **a** ManPDA **b** ManPDA_sr

Figure 6 shows the spatial and temporal patterns corresponding to the extracted filters for supervised MDA PARAFAC-structure models with three components, trained on four of the five CV folds from subject A’s training data. All models extract a waveform similar to the P300 ERP, which is the theoretical foundation of P300 BCI systems. The three components extracted by ManPDA look almost identical, with the characteristic P300 temporal signature and a centrally focused scalp pattern. The component on the right for ManPDA_sr has the same characteristics as the ManPDA components. The logistic regression model for the BCI data was trained to predict the probability of the target class, i.e. the class that should contain the P300 response. The estimated components’ logistic regression coefficients are in line with this since the estimates show that central scalp activity exhibiting the P300-like waveform increases the probability of an observation being from the target class.

The two components shown on the left for ManPDA_sr are difficult to interpret since their spatial patterns are not smooth and their temporal patterns are very high frequent. These two components might represent random noise in the data. Hence, we would not expect these components to contribute to classification performance. This is aligned with the observation from Fig. 3 that classification performance is not improved substantially for higher component numbers.

## Discussion

We saw that supervising the feature extraction step resulted in better classification rates. When feature extraction is not supervised, some directions of the data space that contain class-discriminative information but have low variance, and so explain only a small data proportion, may be lost since unsupervised feature extraction focuses on data directions that best explain data variance. Even when including a large number of components, the unsupervised methods did not obtain competitive classification performances, emphasising the need for supervised feature extraction methods.

Although the manifold optimisation approach obtained substantially higher objective function values than existing heuristic optimisation provides, we did not observe large classification performance differences between the supervised methods on the EEG data. With the same number of components, the Tucker and PARAFAC versions of the methods performed similarly. On the simulated data, though, it was evident that the manifold optimisation approach delivered better classification performance, both for the PARAFAC and Tucker objective functions. Keeping in mind that the simulated data was endowed with a Tucker structure, this seems to indicate that the PARAFAC structured models are robust to deviations from the PARAFAC structure assumption. Both CMDA and the PARAFAC structured manifold methods outperformed the Tucker structured trace-of-ratio manifold method (ManTDA) when low numbers of training observations were used. However, it seems that ManTDA was better able to learn from the available data, overfitting when too few observations were available, but performing better with sufficient training data, whereas the other methods’ performances plateaued and were not able to further improve. Notably, the PARAFAC-versions proposed for MDA are also attractive due to their interpretability.

Combining feature extraction and learning the classifier in one step by BDCA led to the best performance on training data. However, as was also a problem for the ManTDA method, the performance dropped on Stekelenburg&Vroomen test data, especially with many components. This pattern is a sign of overfitting. On BCI data, the performance of ManTDA and BDCA did not drop on the test data as these data sets had substantially more trials.

As was originally recommended, regularising the BDCA methods would probably improve their performance [3]. Regularisation could be done in an unsupervised manner by using a Tucker2 compression of the temporal and spatial modes before applying the supervised methods. The regularisation originally recommended was a smoothing function [3], making the estimated spatial and temporal filters smoother. Alternatively, such a smoothing constraint could be applied to the patterns to make them resemble expressions of neural activity more. Other regularisation options are also possible. For example, L1 or L2 regularisation could be incorporated in the logistic regression model in the BDCA methods.

Likewise, regularisation of the manifold-optimised MDA methods would also protect against the problem with overfitting, that became apparent with the Stekelenburg&Vroomen data. One explanation for the high performance of the existing MDA methods could be that their sub-optimal optimisation induces regularisation, albeit uncontrolled. By optimising over manifolds rigorously, the manner and degree of regularisation can potentially be controlled systematically.

For our manifold optimisation, we used conjugate gradient as provided in the *ManOpt* toolbox [35]. However, more efficient optimisation using newer, more advanced manifold optimisation methods [45, 46] might be beneficial. In order to minimise the amount of pre-processing, we used the raw EEG trial data as input to the compared methods. In view of the lack of pre-processing, the high classification rates are surprising and indicates that the tensor methods are able to extract the temporal, as well as spatial, characteristics of data. Hence, these methods might also be useful for extracting neural phenomena without prior knowledge.

## Conclusion

We set out to investigate whether the performance of Multilinear Discriminant Analysis (MDA) methods could be improved through rigorous optimisation instead of existing optimisation heuristics in the context of single-trial EEG classification. We found that rigorous optimisation does obtain substantially higher objective function values than the existing optimisation procedures. This, however, did not lead to better classification performance on EEG data, while performance improvements were seen on simulated data. Additionally, we compared PARAFAC- and Tucker formulations of objective functions and did not observe large differences between these formulations. However, for model interpretation, we found that the proposed PARAFAC MDA models, which the existing MDA methods do not allow for, are attractive. Finally, we investigate whether it is necessary to use supervised methods when searching for subspaces suitable for classification. Our results showed that supervised feature extraction methods perform substantially better than unsupervised methods, indicating that it is advisable to use observation labels when performing feature extraction. For interpretability, PARAFAC formulations are preferred. Using these methods, we were able to extract spatial and temporal patterns resembling spatial activation and waveforms known to be characteristic of the investigated paradigms. This was achieved without usual pre-processing steps such as filtering. That is, the MDA methods were directed solely by data and the labels of trials when extracting these patterns. Our Matlab implementations are available at https://github.com/laurafroelich/tensor_classification.

## Additional files

Additional file 1 Appendix A - Stationary points. Proof that the stationary points of the trace of matrix ratio and ratio of deteriminants objectives are the same. (PDF 92.7 kb)

Additional file 2 Appendix B - non-uniqueness of the tucker structure. Proof that the Tucker structure leads to non-unique solutions. (PDF 106 kb)

Additional file 3 Appendix C - figures from simulation study. Figures comparing the performances of the different methods on data simulated with three different amounts of noise. (PDF 3318 kb)

## Footnotes

1
http://www.bbci.de/competition/iii/

2
http://www.bbci.de/competition/iii/results/index.html

**Table 1:** Mean letter classification rates for data set II from BCI competition III from the compared methods (top) and best five competition participants (bottom), copied from http://www.bbci.de/competition/iii/results/ index.html

	15 flashes	5 flashes
DATEReig	0.930	0.695
CMDA	0.925	0.695
DATER	0.925	0.670
ManTDA	0.915	0.660
ManPDA	0.910	0.645
BDCA	0.895	0.645
ManPDA_sr	0.890	0.555
BDCATucker	0.890	0.655
ManTDA_sr	0.880	0.555
Tucker2	0.605	0.260
DGTDA	0.595	0.320
Parafac	0.315	0.105
Tucker	0.300	0.090
Parafac2	0.025	0.005
Alain Rakotomamonjy	0.965	0.735
Li Yandong	0.905	0.550
Zhou Zongtan	0.900	0.595
Ulrich Hoffmann	0.895	0.530
Lin Zhonglin	0.875	0.575
