# Supplementary material for: Rigorous optimisation of multilinear discriminant analysis with Tucker and PARAFAC structures
Source: BMC Bioinformatics. 2018 May 30;19:197. doi: 10.1186/s12859-018-2188-0 (PMC5977741; doi:10.1186/s12859-018-2188-0)
Supplement: Supplementary file 1 — Appendix A - Stationary points. Proof that the stationary points of the trace of matrix ratio and ratio of deteriminants objectives are the same. (PDF 92.7 kb) [file 12859_2018_2188_MOESM1_ESM.pdf]

Appendix A
Stationary points

We now show that the stationary points of the trace of matrix ratio objective and ratio of deteriminants objectives are the same. We do this by finding the derivatives of both objective functions, setting them equal to zero, and solving for the projection matrix $\mathbf{U}_{LDA}$.

We find the derivative of the trace of matrix ratios as:

$$\frac{\partial Tr((\mathbf{U}_{LDA}^\top \mathbf{W}_{LDA}\mathbf{U}_{LDA})^{-1}\mathbf{U}_{LDA}^\top \mathbf{B}_{LDA}\mathbf{U}_{LDA})}{\partial \mathbf{U}_{LDA}} = -2\mathbf{W}_{LDA}\mathbf{U}_{LDA}(\mathbf{U}_{LDA}^\top \mathbf{W}_{LDA}\mathbf{U}_{LDA})^{-1} \times \mathbf{U}_{LDA}^\top \mathbf{B}_{LDA}\mathbf{U}_{LDA}(\mathbf{U}_{LDA}^\top \mathbf{W}_{LDA}\mathbf{U}_{LDA})^{-1} + 2\mathbf{B}_{LDA}\mathbf{U}_{LDA}(\mathbf{U}_{LDA}^\top \mathbf{W}_{LDA}\mathbf{U}_{LDA})^{-1}.$$

Setting this equal to zero, we find:

$$\begin{aligned} 0 =& -2\mathbf{W}_{LDA}\mathbf{U}_{LDA}(\mathbf{U}_{LDA}^\top \mathbf{W}_{LDA}\mathbf{U}_{LDA})^{-1} \\ & \mathbf{U}_{LDA}^\top \mathbf{B}_{LDA}\mathbf{U}_{LDA}(\mathbf{U}_{LDA}^\top \mathbf{W}_{LDA}\mathbf{U}_{LDA})^{-1} \\ & + 2\mathbf{B}_{LDA}\mathbf{U}_{LDA}(\mathbf{U}_{LDA}^\top \mathbf{W}_{LDA}\mathbf{U}_{LDA})^{-1} \\ \Leftrightarrow & \mathbf{W}_{LDA}\mathbf{U}_{LDA}(\mathbf{U}_{LDA}^\top \mathbf{W}_{LDA}\mathbf{U}_{LDA})^{-1} \\ & \times \mathbf{U}_{LDA}^\top \mathbf{B}_{LDA}\mathbf{U}_{LDA} \\ =& \mathbf{B}_{LDA}\mathbf{U}_{LDA}. \end{aligned}$$

Set $g_w(\mathbf{U}_{LDA}) = \det(\mathbf{U}_{LDA}^\top \mathbf{W}_{LDA}\mathbf{U}_{LDA})$ and $g_b(\mathbf{U}_{LDA}) = \det(\mathbf{U}_{LDA}^\top \mathbf{B}_{LDA}\mathbf{U}_{LDA})$. From Equation (53), p. 9 in [1], we have:

$$\frac{\partial g_w(\mathbf{U}_{LDA})}{\partial \mathbf{U}_{LDA}} = 2g_w(\mathbf{U}_{LDA})\mathbf{W}_{LDA}\mathbf{U}_{LDA} \times (\mathbf{U}_{LDA}^\top \mathbf{W}_{LDA}\mathbf{U}_{LDA})^{-1}.$$

For the ratio matrix determinants objective, we then find:

$$\begin{aligned} & \frac{\partial \frac{g_b(\mathbf{U}_{LDA})}{g_w(\mathbf{U}_{LDA})}}{\partial \mathbf{U}_{LDA}} = \\ & 2g_b(\mathbf{U}_{LDA})\mathbf{B}_{LDA}\mathbf{U}_{LDA} \\ & \times (\mathbf{U}_{LDA}^\top \mathbf{B}_{LDA}\mathbf{U}_{LDA})^{-1} \frac{1}{g_w(\mathbf{U}_{LDA})} \\ & - \frac{g_b(\mathbf{U}_{LDA})}{g_w(\mathbf{U}_{LDA})^2} 2g_w(\mathbf{U}_{LDA})\mathbf{W}_{LDA}\mathbf{U}_{LDA} \\ & \times (\mathbf{U}_{LDA}^\top \mathbf{W}_{LDA}\mathbf{U}_{LDA})^{-1} \\ & = 2\frac{g_b(\mathbf{U}_{LDA})}{g_w(\mathbf{U}_{LDA})}(\mathbf{B}_{LDA}\mathbf{U}_{LDA}(\mathbf{U}_{LDA}^\top \mathbf{B}_{LDA}\mathbf{U}_{LDA})^{-1} \\ & - \mathbf{W}_{LDA}\mathbf{U}_{LDA}(\mathbf{U}_{LDA}^\top \mathbf{W}_{LDA}\mathbf{U}_{LDA})^{-1}). \end{aligned}$$

We set this equal to zero and solving for $\mathbf{U}_{LDA}$:

$$\begin{aligned} 0 =& 2\frac{g_b(\mathbf{U}_{LDA})}{g_w(\mathbf{U}_{LDA})}(\mathbf{B}_{LDA}\mathbf{U}_{LDA}(\mathbf{U}_{LDA}^\top \mathbf{B}_{LDA}\mathbf{U}_{LDA})^{-1} \\ & - \mathbf{W}_{LDA}\mathbf{U}_{LDA}(\mathbf{U}_{LDA}^\top \mathbf{W}_{LDA}\mathbf{U}_{LDA})^{-1}) \\ \Leftrightarrow & \mathbf{W}_{LDA}\mathbf{U}_{LDA}(\mathbf{U}_{LDA}^\top \mathbf{W}_{LDA}\mathbf{U}_{LDA})^{-1} \\ =& \mathbf{B}_{LDA}\mathbf{U}_{LDA}(\mathbf{U}_{LDA}^\top \mathbf{B}_{LDA}\mathbf{U}_{LDA})^{-1} \\ \Leftrightarrow & \mathbf{W}_{LDA}\mathbf{U}_{LDA}(\mathbf{U}_{LDA}^\top \mathbf{W}_{LDA}\mathbf{U}_{LDA})^{-1} \\ & \times (\mathbf{U}_{LDA}^\top \mathbf{B}_{LDA}\mathbf{U}_{LDA}) \\ =& \mathbf{B}_{LDA}\mathbf{U}_{LDA}. \end{aligned}$$

This is the same equality as before, thus the stationary points are equivalent.

References

[1] K. B. Petersen and M. S. Pedersen, "The matrix cookbook," nov 2012, version 20121115.
