# Supplementary material for: Rigorous optimisation of multilinear discriminant analysis with Tucker and PARAFAC structures
Source: BMC Bioinformatics. 2018 May 30;19:197. doi: 10.1186/s12859-018-2188-0 (PMC5977741; doi:10.1186/s12859-018-2188-0)
Supplement: Supplementary file 2 — Appendix B - non-uniqueness of the tucker structure. Proof that the Tucker structure leads to non-unique solutions. (PDF 106 kb) [file 12859_2018_2188_MOESM2_ESM.pdf]

APPENDIX B
NON-UNIQUENESS OF THE TUCKER STRUCTURE

MDA based on the Tucker structure is not unique when considering the objective functions given above. In fact, the projection matrix for each mode can separately be multiplied by any orthonormal matrix $\boldsymbol{R}$ without changing the value of the objective function.

To see this, consider the scatter ratio objective, where we have numerator and denominator terms of the following structure: $Tr\left(\mathbf{U}_s^\top \mathbf{M} \mathbf{U}_s\right) = Tr\left(\mathbf{U}_s \mathbf{U}_s^\top \mathbf{M}\right)$. Without loss of generality we let $P = 2$. Let $\mathbf{U} = \mathbf{U}^{(2)} \otimes \mathbf{U}^{(1)}$ and $\tilde{\mathbf{U}} = (\mathbf{U}^{(2)}\mathbf{R}) \otimes \mathbf{U}^{(1)}$. We then obtain:

$$\tilde{\mathbf{U}}\tilde{\mathbf{U}}^\top = (\mathbf{U}^{(2)}\mathbf{R}\mathbf{R}^\top \mathbf{U}^{(2)^\top}) \otimes (\mathbf{U}^{(1)}\mathbf{U}^{(1)^\top}) = \mathbf{U}\mathbf{U}^\top,$$

where we have made use of the facts that $(\mathbf{A} \otimes \mathbf{B})^\top = \mathbf{A}^\top \otimes \mathbf{B}^\top$ and $(\mathbf{A} \otimes \mathbf{B})(\mathbf{C} \otimes \mathbf{D}) = (\mathbf{AC} \otimes \mathbf{BD})$ [1].

Again considering $P = 2$ without loss of generality, we obtain for the trace of matrix ratio objective:

$$\begin{aligned}
&Tr\left((\tilde{\mathbf{U}}^\top \mathbf{W} \tilde{\mathbf{U}})^{-1} \tilde{\mathbf{U}}^\top \mathbf{B} \tilde{\mathbf{U}}\right) \\
&= Tr\big(((\mathbf{R}^\top \otimes \mathbf{I}_{K_1})\mathbf{U}^\top \mathbf{W} \mathbf{U} (\mathbf{R} \otimes \mathbf{I}_{K_1}))^{-1} \\
&\quad ((\mathbf{R}^\top \otimes \mathbf{I}_{K_1})\mathbf{U}^\top \mathbf{B} \mathbf{U} (\mathbf{R} \otimes \mathbf{I}_{K_1}))\big) \\
&= Tr\big(((\mathbf{R} \otimes \mathbf{I}_{K_1})^{-1}(\mathbf{U}^\top \mathbf{W} \mathbf{U})^{-1}(\mathbf{R}^\top \otimes \mathbf{I}_{K_1})^{-1}) \\
&\quad (\mathbf{R}^\top \otimes \mathbf{I}_{K_1})\mathbf{U}^\top \mathbf{B} \mathbf{U} (\mathbf{R} \otimes \mathbf{I}_{K_1})\big) \\
&= Tr\left((\mathbf{U}^\top \mathbf{W} \mathbf{U})^{-1} \mathbf{U}^\top \mathbf{B} \mathbf{U}\right),
\end{aligned}$$

where $\mathbf{I}_{K_1}$ is the $K_1 \times K_1$ identity matrix. Here, we have also uased the fact that $(\mathbf{A} \otimes \mathbf{B})^{-1} = (\mathbf{A}^{-1} \otimes \mathbf{B}^{-1})$ [1].

REFERENCES

[1] K. B. Petersen, M. S. Pedersen et al., "The matrix cookbook," Technical University of Denmark, vol. 7, p. 15, 2008.
