# Supplementary material for: Rigorous optimisation of multilinear discriminant analysis with Tucker and PARAFAC structures
Source: BMC Bioinformatics. 2018 May 30;19:197. doi: 10.1186/s12859-018-2188-0 (PMC5977741; doi:10.1186/s12859-018-2188-0)
Supplement: Supplementary file 3 — Appendix C - figures from simulation study. Figures comparing the performances of the different methods on data simulated with three different amounts of noise. (PDF 3318 kb) [file 12859_2018_2188_MOESM3_ESM.pdf]

APPENDIX C
FIGURES FROM SIMULATION STUDY

Figures 1, 2, and 3 show simulation results from simulated data with low, medium, and high signal strength, respectively. Each curve is the mean of AUCs over 25 simulations, and the black vertical lines show plus/minus one standard deviation of the mean.

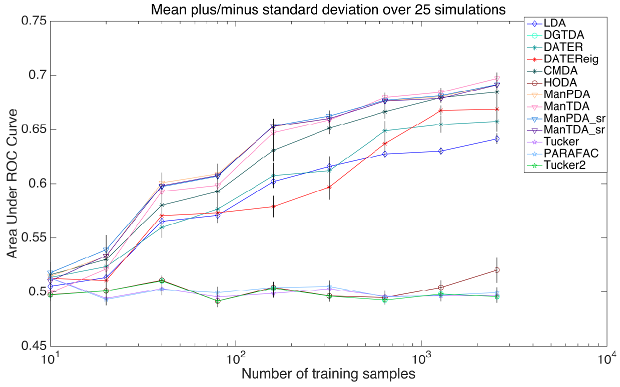

Figure 1. AUCs obtained by the tensor decomposition methods when low signal strength is simulated.

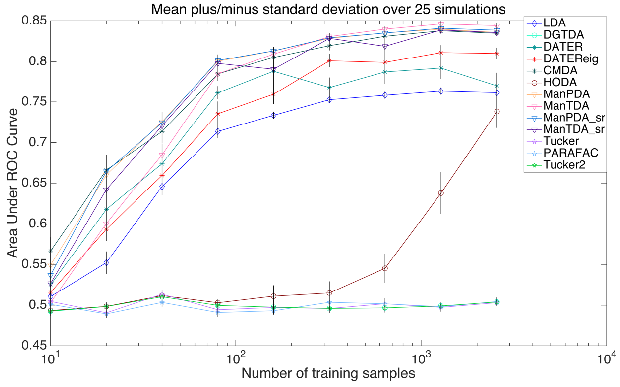

Figure 2. AUCs obtained by the tensor decomposition methods when medium signal strength is simulated.

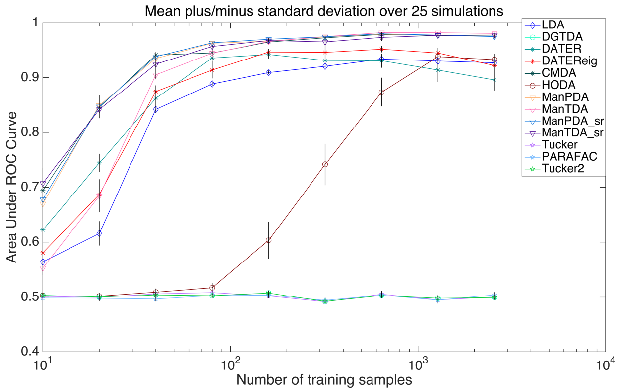

Figure 3. AUCs obtained by the tensor decomposition methods when high signal strength is simulated.
